# Ethical Impact Assessment of Sharing Nanosafety Data

**DOI:** 10.12688/openreseurope.18345.2

**Published:** 2026-04-06

**Authors:** Ineke MALSCH, Egon Willighagen, Candida F. Sánchez Burmester, Cyrus Mody

**Affiliations:** 1 Malsch TechnoValuation, Utrecht, PO Box 455, 3500 AL, The Netherlands; 2 Translational Genomics, Universiteit Maastricht School of Nutrition and Translational Research in Metabolism, Maastricht,Universiteitssingel 40, Limburg, 6229 ER, The Netherlands; 3 Maastricht University Faculty of Arts and Social Sciences, Maastricht, Limburg, Grote Gracht 90-92 6211 SZ, The Netherlands

**Keywords:** Nanosafety data, FAIR data, open access, ethics, health, liberties, equality, common good, environment, misuse

## Abstract

Decades of research on environmental, health and safety impacts of nanomaterials have generated large amounts of nanosafety data, but not all data are available online following the Findable, Accessible, Interoperable, and Reusable (FAIR) principles. This lack of FAIR data delays progress in regulatory research, needed by regulators to develop evidence-based policies. In this context, researchers collaborating in the EU NanoSafety Cluster ( www.nanosafetycluster.eu) engaged in a discussion on the ethical impacts related to (not) sharing nanosafety data. Screening the potential ethical impacts suggested that *openly sharing nanosafety data relevant to risk governance of nanomaterials could give rise to ethical issues related to health and to liberties, and that these data might be misused.* On the other hand, not *openly sharing such nanosafety data could raise issues related to health, equity, common good, environment, and sustainability.* A small-scale Ethical Impact Assessment (EIA) was performed between June 2022 and April 2024 to identify and evaluate more specific ethical issues and to propose recommendations for remediating ethical concerns. Preliminary results were presented in scientific conferences (one poster and three oral presentations), while the comments of the participants were incorporated in the final version of the presented paper. The EIA identified ethical issues and value conflicts related to the following sectors: intellectual property, social justice, public health, dual use, environmental ethics and animal ethics. Some recommendations for remediation are the competence of research organisations. Other recommendations call for societal dialogue and engagement. Yet other recommendations call for political action.

## Plain language summary

Researchers in public and private research organisations have been investigating the risks of nanomaterials for human health and the environment for decades. The results of these studies are needed to support risk governance of these nanomaterials and the products they are applied in, and to enable their evidence based regulation. Not all data resulting from these studies is published in a Findable, Accessible, Interoperable and Reproducible (FAIR), or open access format. This article reports on an Ethical Impact Assessment (EIA) study exploring ethical issues raised by decisions to either publish all data open access or not to publish (all) data. Ethical issues and value conflicts related to intellectual property, social justice, public health, dual use, environmental ethics and animal ethics were identified. Preliminary findings were discussed with participants in scientific events, and their responses were used to improve the analysis and recommendations. The article concludes with recommendations for research organisations, stakeholders participating in societal dialogue and engagement, and politicians.

## Introduction

The use of nanomaterials has increased over the last two decades, especially in cosmetics, drugs, medicine, food, water treatment, soil conservation, microelectronics, and energy production. These materials are often praised for their properties, but it is equally important to examine their potential toxicity and risks to humans, animals and the environment. Risks *and* benefits may be associated *both* with the properties of novel materials themselves, and with the novel ways of organizing and conducting science needed to research those properties. This article examines the ethics of new practices in nanotoxicology in part to facilitate more effective and responsible understanding of the risks from novel nanomaterials.

In terms of *practices*, nanosafety researchers have adopted existing methods from toxicology, but the unique characteristics of nanomaterials also require new approaches. These new approaches are used to investigate a) if engineered nanomaterials such as quantum dots, carbon nanotubes, and graphene are toxic, and b) if there is toxicity that is specific to the size and large surface area of nanoparticles, especially in comparison to the same material in bulk form. An important element in nanosafety research is the life cycle assessment (LCA) of nanomaterials, evaluating the sustainability and environmental impact of these materials from their production to their disposal ( Lebre *et al.*, 2022).

Recently, *in silico* nanosafety assessment tools have been used more widely to study data-driven toxicity prediction of nanomaterials, fate modelling for environmental pollution, and modelling of nano-bio interactions for long-term effects. The goal of *in silico* assessment tools is to investigate which physicochemical parameters of engineered nanomaterials could lead to toxic mechanisms in a given biological environment ( Bansod *et al*., 2025). The physicochemical parameters of engineered nanomaterials involve intrinsic properties (e.g., shape, porosity, and bandgap), extrinsic properties (e.g., concentrations and surface interactions with biomolecules), and composition aspects (e.g., charge, hydrophobicity, and coating) ( Hassan & Singh, 2014). Thus, regarding risks associated with the materials themselves, the four recognized toxicity mechanisms of nanomaterials are 1) the release of toxic chemical substances from nanomaterials, 2) direct effects from physical contact with nanomaterials resulting in interference with biological functions, 3) inherent properties of the nanomaterials (e.g., photochemical), and 4) the crossing of biological barriers and translocation to sensitive tissues due to their size. In order to investigate correlations between the nanomaterials’ physicochemical parameters, the biological environment, and the toxicity mechanisms, large amounts of data are needed. Therefore, data-sharing practices and databases are a crucial requirement for *in silico* nanosafety assessment ( Yu *et al.*, 2021).

RiskGONE is one of three projects funded by the EU under the Horizon 2020 programme from 2019 until 2023, aiming to develop new guidelines to assess the risk of nanomaterials. The sister projects are Gov4Nano and NanoRIGO. Info: https://riskgone.eu/ or https://cordis.europa.eu/project/id/814425. Partners in this H2020 RiskGONE projecton Risk Governance of Nanomaterials ( Isigonis *et al.*, 2020) discussed the statement “It is unethical not to share [nanosafety] data [needed by other researchers and for regulation]”. During the consortium meeting of the project in Limassol (Cyprus) on 24 June 2022, the issue was briefly discussed. The lead author is a professional ethicist; from that perspective, despite the RiskGONE partners’ instinctive framing, it is not obvious that this question should be discussed in ethical terms. One possible reason for introducing ethics, however, could be that the current regulations are not sufficient for enforcing the sharing of Open or at least Findable, Accessible, Interoperable and Reusable (FAIR) data ( Nature, 2016). In addition, researchers may not be sufficiently rewarded for sharing FAIR data. Different philosophical ethical theories are used to analyse ethical issues and dilemmas, but professional ethicists tend to make a distinction between ethical analysis and moral arguments used by stakeholders in discussions on what should be done in a particular case. For example, deontological ethical theories stress obligations to comply with predetermined rules ( Alexander & Moore, 2022), while consequentialism puts more emphasis on beneficial outcomes of actions ( Sinnott-Armstrong, 2023). From a virtue ethics perspective, individuals should let their personal values, such as solidarity, justice, or honesty, guide their actions, even when they are not legally obliged to undertake them and also if the actions go against their personal interests ( Hursthouse & Pettigrove, 2023, see also Steen *et al.*, 2021, who apply virtue ethics to responsible innovation).

However, the question whether or not nanosafety data should be shared openly is subject to conflicting values. Values favouring data-sharing include contribution to the common good ( Kohonen-Corish *et al.*, 2010), sustainability ( Kostal *et al.*, 2022) or transparency ( Boué *et al.*, 2018). Values favouring restrictions on data-sharing include a lack of quality or validity of specific data ( Krug, 2018), legitimate ownership of (one’s own or other actor’s) data ( Briggs *et al.*, 2021), and avoiding misuse by others ( Bezuidenhout, 2013). Related to civil liberties, in some cases, sharing nanosafety data could infringe on the intellectual property rights of data owners. Thus, a simple ethical stance was not immediately apparent, and at the end of the discussion in Limassol, it was suggested to perform a full Ethical Impact Assessment (EIA) to delve deeper into the ethical concepts and values at stake, and further discuss the findings with RiskGONE partners.

Related to the previous question is the consideration of what kind of nanosafety data should be shared. Scientists from different laboratories have suggested the Minimum Information Reporting in Bio–Nano Experimental Literature (MIRIBEL), including the characterisation of nanomaterials, characterisation of biological environments such as information on cell seeding and cell characterisation, and details of experimental protocols ( Faria *et al*., 2018). A group of researchers collaborating in the Nanosafety Cluster, including members of the RiskGONE project, have addressed this issue. Health-related ethical risks of sharing nanosafety data could emerge if insufficiently verified nanosafety data are used, which might be wrongly interpreted and indirectly contribute to health risks, environmental impacts and false claims that might circulate in the scientific record and/or in more public-facing media.

Health-related ethical benefits could also emerge, since publishing sound nanosafety data is expected to contribute to limiting health risks of nanomaterials. Regarding equality, health inequalities of workers and consumers exposed to nanomaterials are expected to be reduced if sound nanosafety data are used to improve regulation. Concerning the common good, swift access to sound nanosafety data may enable evidence-based regulation of nanomaterials. Furthermore, publishing sound nanosafety data is expected to contribute to limiting environmental impacts of nanomaterials, and to Sustainable Development Goal (SDG) 12 on sustainable consumption and production ( Alfaro Serrano *et al.*, 2024).

**Figure 1.**
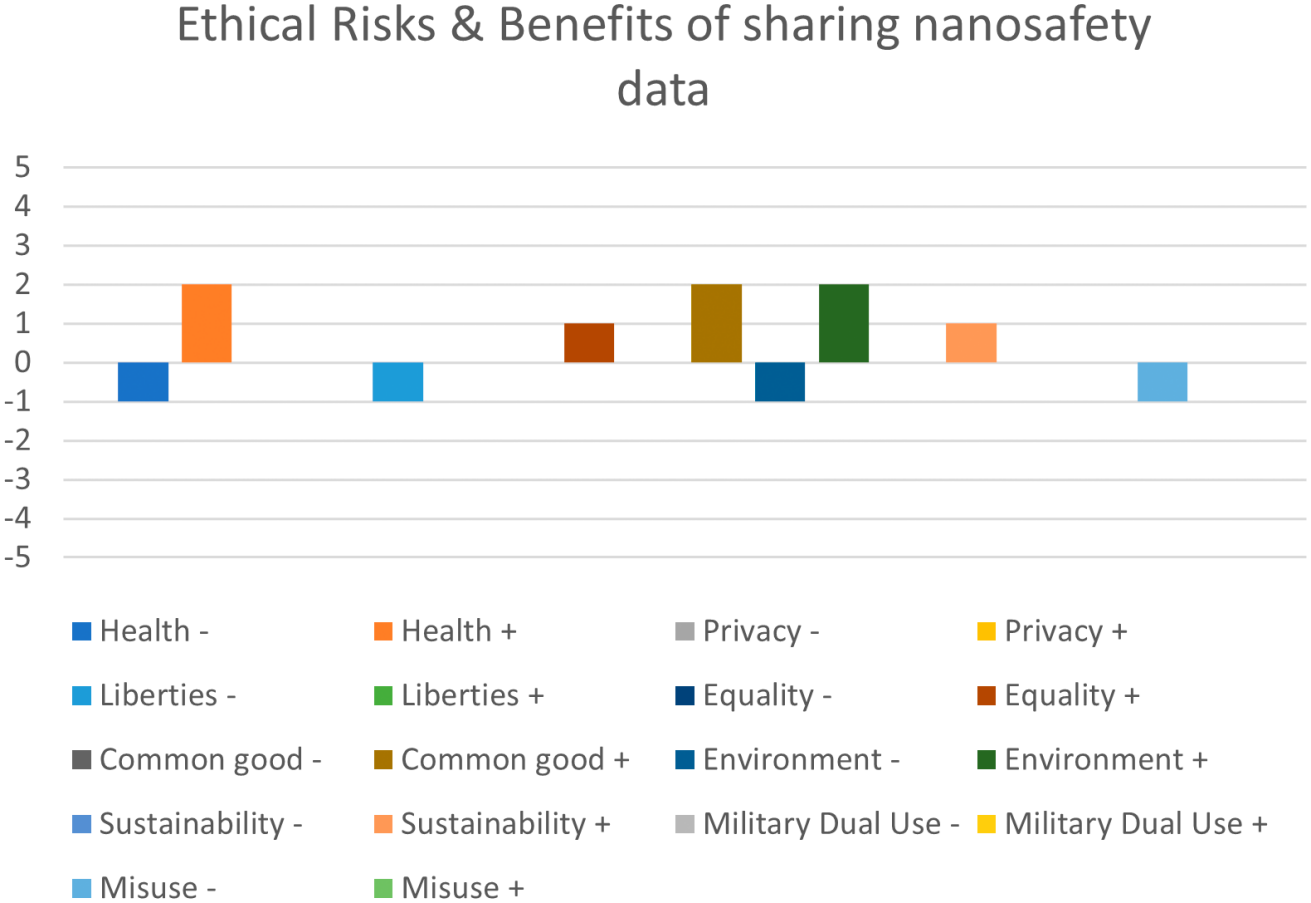
Ethical risk-benefit screening of sharing nanosafety data.

Figure 1 presents the output of a risk-benefit assessment of sharing nanosafety data. A screening of the ethical issues was performed with the use of the RiskGONE Ethical Impact Assessment screening tool ( https://www.enaloscloud.novamechanics.com/riskgone/EIA/thresholdanalysis/index.zul), published open access under creative commons licence CC BY 4.0 at the ENALOSCLOUD platform of NovaMechanics: https://www.enaloscloud.novamechanics.com/.

The result of the screening of ethical issues suggested that a small EIA should be performed for this study. The results of this small EIA are presented in this paper, answering the questions:

1) Which ethical issues and value conflicts are at stake in decision-making regarding openly sharing nanosafety data needed for risk governance of nanomaterials?
2) How can these issues be resolved?
3) Should nanosafety data be published or not?
4) When should nanosafety data (not) be published and how should it (not) be published?

## Methods

The six-step EIA procedure was based on the CEN Workshop Agreement 17145–2:2017 ( CEN, 2017), and supported by the Ethical Impact Assessment guidelines ( RiskGONE D3.6, 2020) and online tools developed in the RiskGONE project ( https://www.enaloscloud.novamechanics.com/riskgone/EIA/, Isigonis *et al.*, 2020; Malsch *et al.*, 2020). The methodology of the RiskGONE EIA guidelines and the related online tools are explained more in depth in Malsch *et al.* (2024), where the outputs of a number of case studies related to nanosafety are also presented and compared. Readers who want to learn more about the methodology and in which way the CEN Workshop Agreement was adapted to risk governance of nanomaterials are encouraged to read that paper first.

The work reported in the poster “Ethical Impacts of (Not) Sharing Nanosafety Data” ( Malsch *et al.*, 2023) is included in that comparison. The present paper concentrates primarily on the identified issues, evaluation of relevant values, and stakeholder engagement (i.e., dialogue with those affected by RiskGONE’s decisions about the sharing of its data) in preparing recommendations for remediation. Below, the six-step EIA procedure applied to the case of sharing nanosafety data is briefly described.

Step 1 consisted of screening ethical impacts with a simple self-assessment tool, asking the user of the online tools ( https://www.enaloscloud.novamechanics.com/riskgone/EIA/thresholdanalysis/index.zul) to indicate the relevance of nine ethical categories, and to estimate the severity of expected ethical impacts (NO = no impact, 1 = minor, 2 = moderate, 3 = medium, 4 = strong, 5 = severe). The severity of ethical impacts can be measured in four dimensions: the frequency of occurrence, the number of people who may be affected, the duration and reversibility of the impact, and the additionality to existing impacts (see Text Box 1).

Text Box 1: **To what extent will the publication of nanosafety data give rise to the following issues?**

No = I don’t think it is applicable. The publication of these nanosafety data has no effect on this ethical issue.

1 = I foresee minor ethical impacts on this issue. The effects are very unlikely and/or have very low potential strength – examples: the frequency of this ethical impact is less than once in 100 years, or only few people are likely to be affected temporarily and reversibly, or the new technology does not significantly worsen or improve ethical impacts caused by existing alternatives.

2 = I foresee moderate ethical impacts on this issue. The effects are unlikely and/or have mild potential strength - examples: this ethical impact may happen a few times in a lifetime, or few people are likely to be affected mildly for a long time or irreversibly, or many people are likely to be affected temporarily and reversibly, or the new technology noticeably worsens or improves ethical impacts caused by existing alternatives.

3 = I foresee medium ethical impacts on this issue. The effects are substantially likely and/or have substantial potential strength – examples: this ethical impact is expected to occur several times in a lifetime, or at least a few people are likely to be affected substantially for a long time or irreversibly, or the new technology substantially worsens or improves ethical impacts caused by existing alternatives.

4 = I foresee strong ethical impacts on this issue. The effects are likely and/or have high potential strength: this ethical impact will occur frequently, or many people are likely to be affected strongly for a long time or irreversibly, or the new technology seriously worsens or improves ethical impacts caused by existing alternatives.

5 = I foresee extreme ethical impacts on this issue. The effects are very likely and/or have very high potential strength – examples: the high ethical impact occurs permanently, or many people are affected strongly and irreversibly, or (if negative) fundamental human rights are violated, or large scale societal disruption is expected.

The nine categories of ethical issues assessed were: health-related, privacy, liberties, equality, common good, environment, sustainability, military dual use and misuse. The output of the tool was an advice on the need to perform no EIA, or a small-, medium- or large-scale EIA. In this case, a small scale EIA appeared appropriate to analyse the relevant ethical issues. In step 2, the ethicist drafted an EIA plan. The required expertise to perform the small-scale EIA included: ethics, LCA, informatics, software programmers, risk assessment, nanosafety, risk governance. The EIA was reviewed by two co-authors of this paper: an historian of recent science and technology and a researcher in Science and Technology Studies (STS), both of whom are familiar with responsible innovation issues relating to nanotoxicology, taking the following criteria into account: relevance of research questions and resources to address the ethical issues identified during pre-screening, salience of results and outcomes.

Step 3 consisted of identifying the relevant ethical issues. The ethicist performed desk research to identify ethical issues in similar cases in literature available online. The literature review had a clear plan and followed best practices. The search followed the methodology of a Systematic Review (O’Connor *et al.*, 2012). The selection process of the literature review started with a search with different combinations of keywords including ‘nanosafety data’, ‘sharing data’ and ‘ethics of sharing data’. In addition to generic searches, articles published in specialised journals including the *Data Science Journal*, *Nanoethics*, *Science and Engineering Ethics*, *Biomed Central* (BMC) and other Springer publications, *Journal of Responsible Innovation*, *University World News*, and STS-conference Graz proceedings were searched. Furthermore, websites and repositories of organisations issuing ethical guidelines, regulations or recommendations for nanotechnology, ICT or scientific publishing were searched, including UNESCO (2017, 2021), National Academies of Sciences, Engineering and Medicine (USA), the Budapest Open Access Initiative (2002), European Commission, the European Group on Ethics in Science and New Technologies to the European Commission, European Group on Ethics, 2012 & European Group on Ethics, 2015, the Committee on Publication Ethics, Knowledge Rights 21, ISO TC 229 Nanotechnologies, Science Europe (2022), SAICM, UNEP, and OECD. This was followed by snowballing, checking relevant references in identified sources. Comments by co-authors and external stakeholders also revealed additional literature which was added to the analysed references at a later stage.

Given the fact that nanosafety data are mainly limited to results of (eco) toxicity testing and other risks assessment studies, where personal data of human data owners are not routinely collected or stored, cases focusing narrowly on privacy and personal data protection were not considered similar. Cases addressing chemical data, open science, big data and AI were considered similar. The literature review covered both nanosafety publications discussing ethics of data-sharing and the open science movement more generally, especially ethical frameworks such as codes of conduct or discussions of regulations and responsible research and innovation.

In step 4, the ethicist applied ethical theories and concepts to analyse the identified issues. Step 5 consisted of drafting and discussing recommendations. The ethicist drafted remediation recommendations. The identified issues, draft outcomes of the analysis and proposed recommendations were discussed with researchers during the XX Seminar on Nanotechnology, Society and Environment in Brazil and online, on 18 October 2023( https://www.nanotecnologiadoavesso.org/content/xx-semin%C3%A1rio-internacional-nanotecnologia-sociedade-e-meio-ambiente-e-xx-semana-nacional-de-). Comments from participants in the Bioinformatics in Action seminar at Maastricht University on 11 January 2024 ( https://www.maastrichtuniversity.nl/research/bioinformatics/events/BiA) were also incorporated. The recommendations included actions to be performed by Nanosafety Cluster projects, as well as policy recommendations. Finally, in step 6, external journal peer reviewers reviewed the manuscript during the open peer review process of Open Research Europe.

## Identification of ethical impacts

Scientific literature available online in selected journals was collected and analysed with a more extensive checklist ( http://www.enaloscloud.novamechanics.com/riskgone/EIA/idethimpact/index.zul) than the one used in the screening step. The ethical impacts of sharing nanosafety data were identified and also the ethical impacts of not sharing nanosafety data. This resulted in the following report on the identified ethical impacts. The analysis and identification of ethical impacts led to the listing of twelve issues: five related to sharing nanosafety data and seven related to not sharing data. The ‘Ethical impacts of sharing nanosafety data’ section reports on five issues. The ‘Ethical impacts of not sharing nanosafety data’ section reports on the other seven issues. The section ‘Comparison with stakeholders’ assessment reports on the evaluation of the twelve issues by the fifteen scientists in the XX Seminar on Nanotechnology, Society and Environment in Brazil, and on issues discussed with data scientists at the Bioinformatics in Action seminar at Maastricht University.

### Ethical impacts of sharing nanosafety data

While sharing nanosafety data does not raise categorical ethical issues, on a case-by-case basis, open access to some nanosafety data in a particular context may give rise to ethical impacts, estimated by experts to range from minor (1) to moderate (2) in comparison to other ethical issues of science and technology discussed in the ethics literature.

Moderate infringements on intellectual property rights, affecting the rights and liberties of some researchers may occur when researchers in resource-poor settings are pressured into sharing their hard-won nanosafety data. For example, South African researchers were concerned that sharing research data could make them lose the scientific edge ( Bangani & Mojo, 2019). On a scale of 1–5 (see Text Box 1), the ethicist estimates the effects to have mild potential strength (2). When scientists from the Global South and other resource-poor laboratories are required to openly share the data they have produced with a lot of effort, they have significantly less time for further analysis of these data than previously, when results were shared with scientific peers in academic literature without necessarily giving them access to all research data. The negative consequences can be remediated, and the disadvantage is not much stronger with the requirement to share than without it.

Sharing nanosafety data could furthermore have a moderate negative impact in terms of social justice and equality in two ways. First, the distribution of economic resources may be unbalanced to the disadvantage of some researchers, as long as it is not clear who pays for open access and for data storage. Will freelance researchers or researchers in resource-poor settings have the same opportunities to publish their results when authors are required to pay the costs of making their publication Open Access in their preferred journal? In particular, effects on individuals, groups and communities in countries in the Global South are foreseen: Researchers in resource-poor settings in countries in the Global South expect to be disadvantaged even more if they have to share the research data that they collected, given that researchers in such countries usually have to work more slowly and with less advanced equipment than peers in better-resourced labs ( Bangani & Mojo, 2019; Bezuidenhout, 2017). The same applies to less-resourced laboratories in industrialised countries. The effects have mild potential strength, because many people (disadvantaged researchers) are likely to be affected temporarily and reversibly, provided that governments and well-resourced research institutions and peers invest in measures to level the playing field. In addition, the impacts of sharing nanosafety data are not altogether detrimental for less-resourced researchers, because open-source data may also improve these researchers’ opportunities to access data from better-resourced laboratories.

Moreover, moderate impacts may occur by sharing some dual-use data: Some nanosafety data could be misused for biological or chemical weapons ( Bezuidenhout & Morrison, 2016; Bangani & Mojo, 2019). The effects are unlikely under current nanosafety research practices, because it not only depends on the public availability of the data, but also on the presence of educated experts able to interpret the data having harmful intentions. As other concerns about chemical and biological weapons, such misuse is subject to bio- and chemical security measures under binding (inter) national law. A constant concern during review conferences of the Biological and Chemical Weapons conventions is monitoring progress in science and technology which could impact the protective force of these conventions and other measures in the international web of prevention. Example,s of this web of prevention are: the Scientific Advisory Body (SAB) of the Organisation for the Prohibition of Chemical Weapons reports on emerging technologies influencing the implementation of the Chemical Weapons Convention: https://www.opcw.org/about/subsidiary-bodies/scientific-advisory-board and progress in Science and Technology is a standing agenda item during meetings of the Biological and Toxin Weapons Convention: https://disarmament.unoda.org/biological-weapons/science-and-technology.

Recently, concerns have been raised regarding the increasing use of AI to interpret big data on safety of nanomaterials and other chemicals, similar to the dual-use risks of artificial-intelligence-powered drug discovery highlighted by Urbina *et al.* (2022) and the European Commission ( European Commission, 2022). At this stage, it is not clear whether and to what extent open sharing of dual-use nanosafety data in combination with the application of AI to interpret this data may increase the risks of misuse of these data in the future. Further research is needed, applying expert engagement and foresight methodologies, which goes beyond the scope of the present article.

The publication of insufficiently verified nanosafety data could also be wrongly interpreted, thereby indirectly contributing to environmental, health and safety risks, and unqualified publication of nanosafety data could be misinterpreted, thereby being used to support false claims ( Bangani & Mojo, 2019; Papadiamantis *et al.*, 2020). This could raise minor public health and safety issues, cause environmental impacts, and some sensitive data could accidentally be misused. The effects are unlikely if the person who wrongly interprets the data does so in good faith and is willing to be convinced by reasonable arguments. However, the risks could be more severe if the data are intentionally misinterpreted to further a political agenda. Since this problem already exists, sharing of nanosafety data is not likely to significantly worsen ethical impacts caused by existing publication practices. Nevertheless, steps should be taken to mitigate the vulnerability of existing publication practices to misinformation campaigns.

Finally, a significant societal risk of sharing nanosafety data is that the costs (in terms of time, money, the researchers’ energy and attention, etc.) of sharing will prevent societally-useful science from being done (see also Mirowski, 2011). Data-sharing practices are time consuming as it can involve filling in ethics forms, waiting for the approval of an ethics board, obtaining consent from research participants in case human data owners are involved, cleaning the dataset, finding a suitable database, specifying the meta-data, etc. These tasks that often come with open science can result in a workload creep, where researchers are expected to do more, including more superficial work in the same number of paid hours, often at the expense of their research time ( Hostler, 2023).

### Ethical impacts of not sharing nanosafety data

In contrast, the foreseen ethical impacts of not sharing nanosafety data are expected to range from minor to strong (1–4 in a 5-point scale). The effects are discussed in order of relative importance.

Strong effects on the equal distribution of risks and hazards are likely, because health inequalities of workers and consumers exposed to nanomaterials are expected to be reduced if sound nanosafety data are used to improve regulation. The reason is that democratic national governments and the EU must base the binding legislation they adopt on scientific evidence. Since the risks of nanoparticles are potentially significantly different from the risks of larger particles of the same substance ( Kim *et al.*, 2012; Miao *et al.*, 2024), nanosafety data is needed to inform decision making on new or adapted legislation, including laws protecting the health and safety of workers, consumers, and ecosystems. Many people are likely to be affected strongly and data-sharing seriously improves ethical impacts caused by existing publication practices.

Not publishing sound nanosafety data is expected to contribute medium ethical impacts to limiting health risks of nanomaterials. Information disclosure is closely linked to the right to know, one of the basic human rights defined by the United Nations ( UNEP, 2020). Moreover, in the document on principles on human rights and the protection of workers from exposure to toxic substances ( Tuncak, 2019), the Special Rapporteur on toxics and human rights specifies that health and safety information about toxic substances must never be confidential. There is a need to increase transparency by sharing the information needed for the removal of harmful practices while ensuring that confidential business information is well protected. It is imperative to increase understanding of chemicals in commerce and transparency of information on chemicals of concern contained in products to help actors in the value chain make informed decisions and to incentivise action towards the use of safer alternatives ( SAICM, 2022). The effects of sharing sound nanosafety data contributing to the right to know about risks, enabling stakeholders to limit health risks of nanomaterials are likely to occur and have significant potential strength. Open access data-sharing noticeably improves ethical impacts caused by existing publication practices, where not all data are shared publicly.

In addition, medium effects (3 on a scale of 1–5, see Text Box 1) on the well-being and interests of individuals and groups in society, including the quality of work are expected, because undelayed access to sound nanosafety data, once it is appropriately verified, enables evidence-based regulation of nanomaterials. For example, workers manufacturing nanomaterials or products incorporating nanomaterials can be exposed to nanomaterials causing uncertain health risks in the workplace in the absence of binding occupational health and safety legislation. The UN Environment Programme considered that “perhaps the largest gaps in knowledge necessary for regulation and sustainable management of nanomaterials are production, use and end-of-life of nanomaterials” ( UNEP, 2020, p 47). The effects are likely to occur. At least a few people are likely to noticeably suffer from the consequences of inadequate legal protection due to delayed sharing or a lack of sharing for a long time. In addition, data-sharing significantly improves ethical impacts caused by existing publication practices, where not all data are published.

Related to this, not sharing nanosafety data is expected to have medium effects on replicating and building upon existing research. Publishing nanosafety data, including results, pre-registered reports and protocols, allows scientists to reproduce certain experiments and potentially fill existing gaps which might help a scientific community to advance in a certain topic and/or continue where other scientists have left off. In this way, published nanosafety data would contribute to a more robust scientific record. Related to this, some experts prefer using only open data which may be less complete than data behind paywalls; pervasive data-sharing would reduce that asymmetry. The impacts on replicating and building upon existing research are unclear.

Publishing sound nanosafety data is furthermore expected to contribute moderately (2 on a scale of 1–5, see Text Box 1) to limiting environmental impacts of nanomaterials, released from products containing nanomaterials during manufacturing, use and waste processing. Adoption of binding environmental protection legislation is delayed in the absence of public research data about the environmental fate of released nanomaterials. In addition, not sharing nanosafety data delays the emergence of *in silico* risk assessment and extends the use of animal testing ( Burden *et al.*, 2017; Elberskirch *et al.*, 2022). The effects have mild potential strength. FAIR data-sharing noticeably improves ethical impacts caused by existing publication practices, because FAIR data is more accessible for integration into datasets needed for regulatory research which forms the basis for evidence-based legislation governing risk assessments of nanomaterials before market introduction. Regulators must be convinced of the trustworthiness of ‘New Approach Methods’ replacing animal testing. When FAIR data are shared more openly, the establishment of the evidence basis will be achieved sooner than when not all data are shared.

Likewise, moderate impacts are expected on SDG 12, target 12.4 (2002 Johannesburg World Summit on Sustainable Development): the achievement of the sound management of chemicals throughout their life cycle so that by the year 2020, chemicals are produced and used in ways that minimize significant adverse impacts on the environment and human health. Not sharing nanosafety data will delay the achievement of this goal, because global sound management cannot be achieved without global access to knowledge of the properties of the chemicals that are being managed ( SAICM, 2022, https://sdgs.un.org/goals/goal12). The effects have mild potential strength. Data-sharing noticeably improves ethical impacts caused by existing publication practices. Related to this, not sharing nanosafety data forces others to repeat the experiment. For (pharmaceutical) companies, this is a useful strategy to protect their intellectual property. These well-resourced companies routinely repeat experiments reported in literature to test reproducibility. Nanosafety risks assessment, in contrast, is a small research area with low budgets. Paradoxically, the results of successful nanosafety analysis does not generate more income for researchers in this field. Therefore, open access to sound data is important, to avoid repeating the same costly analyses or studying the same (paywalled) articles. It is worthwhile to develop a database allowing to screen articles on how the included data was subsequently used. Whether this would help speed up progress in the field is a topic for future investigation.

Finally, imposing restrictions on sharing nanosafety data which could be misused by others may negatively affect academic freedom ( Bezuidenhout & Morrison, 2016). This raises minor ethical impacts. The effects have very low potential strength. Only a few people are likely to be affected temporarily and reversibly, and data-sharing does not significantly improve ethical impacts caused by existing publication practices.

### Comparison with stakeholders’ assessments

The preliminary results of the EIA were presented online to participants in the XX Seminar on Nanotechnology, Society and Environment in Brazil, on 18 October 2023. ( Malsch, 2023) After the presentation, fifteen distinct responses to an online survey were received. (Sixteen anonymous responses were received in total, including one exact duplicate of another answer, which was not included in the analysis, because the respondent probably submitted the same reply twice.) On average, all (mean) estimates of the severity of each identified issue were higher than the estimates of the ethicist performing the original assessment (Ineke Malsch). In one case, the ethicist’s assessment was equal to the median of the fifteen stakeholder’s assessments: both the ethicist’s and the median respondent’s rating of the severity of “not sharing nanosafety data prolongs health inequalities” was high. The difference between the mean and median stakeholder and the ethicist’s assessment was most similar (limited to 1 point difference or less) for issues related to health inequalities (1), preventing other useful research (12), and slowing down evidence-based regulation (3). The difference ranged from 1.1 to 2 for issues related to the human right to know about health risks (2), limiting environmental impacts (4), adopting *in silico* testing (5), intellectual property rights (8), competitiveness of less-resourced researchers (9), and misuse potential (10). The difference was 2.1 to 3 for sustainability (6) and academic freedom (7), and the difference was 3.1 to 4 points for issue 11: *Misinterpretation of insufficiently verified nanosafety data could contribute to environmental, health and safety risks, and be used to support false claims* (see Table 1 below). The range between the highest and lowest estimates of stakeholders differed per issue, indicating varying levels of disagreement among them. This range was relatively limited (2 points) for issues 1 and 2, increasing to 3 points for issues 3, 5, 7, 11 and 12, up to 4 points for issues 6, 8 and 10, and reached the maximum on the 5-point scale for issues 4 and 9.

**Table 1.** Comparing survey responses of 15 stakeholders with the estimates of the ethicist performing the EIA for 12 identified issues.

Issue	ethicist	mean	median	range	mean-eth	median-eth
1. Not sharing nanosafety data prolongs health inequalities	4	4,2	4	2	0,2	0
2. Not sharing nanosafety data infringes on the human right to know about health risks	3	4,5	5	2	1,5	2
3. Not sharing nanosafety data slows down evidence based regulation of nanomaterials	3	4,0	4	3	1,0	1
4. Not sharing nanosafety data slows down the limiting of environmental impacts of nanomaterials	2	3,4	4	5	1,4	2
5. Not sharing nanosafety data delays the emergence of in silico risk assessment and extends the use of animal testing	2	3,9	4	3	1,9	2
6. Not sharing nanosafety data delays the sound management of chemicals throughout their life cycle (SDG 12.4)	2	4,1	4	4	2,1	2
7. Restricting sharing nanosafety data which could be misused by others may negatively affect academic freedom	1	4,0	4	3	3,0	3
8. Sharing nanosafety data impacts on intellectual property rights when researchers in resource-poor settings are nudged into sharing hard-won nanosafety data	2	3,7	4	4	1,7	2
9. Researchers in resource poor settings could be disadvantaged if forced to share their data	2	3,1	4	5	1,1	2
10. Some nanosafety data could be misused for biological or chemical weapons	2	3,9	4	4	1,9	2
11. Misinterpretation of insufficiently verified nanosafety data could contribute to environmental, health and safety risks, and be used to support false claims	1	4,5	5	3	3,5	4
12. The costs of sharing nanosafety data could prevent societally-useful science from being done	2	2,9	3	3	0,9	1

The systematic gap between the ethicist’s estimate of the severity of ethical issues and the estimates of stakeholders suggests an underlying difference between interpretations of the scale. The ethicist places the identified ethical issues in the context of the professional ethical knowledge base of ethical impacts of science and technology or human activities in general, where extreme events such as nuclear war or climate change-induced disasters would score 5 points (severe). In comparison, most risks of sharing nanosafety data are minor or moderate. Most stakeholders are not professional ethicists, and tend to use the full five-point scale to rank the severity of identified ethical issues. Therefore, rather than taking the estimates at face value, they should be taken as indicators of issues where deeper analysis of the corresponding ethical issues is needed through expert and stakeholder consultations. Deeper analysis should primarily target issue 11 ( *Misinterpretation of insufficiently verified nanosafety data could contribute to environmental, health and safety risks, and be used to support false claims*), where the difference between the average respondent and the ethicist is more than 3 points. This should be followed by issues 7 ( *Restricting sharing nanosafety data which could be misused by others may negatively affect academic freedom*), and 6 ( *Not sharing nanosafety data delays the sound management of chemicals throughout their life cycle (SDG 12.4)*). In both cases, the difference between the average respondent and the ethicist is more than 2 points.

**Figure 2.**
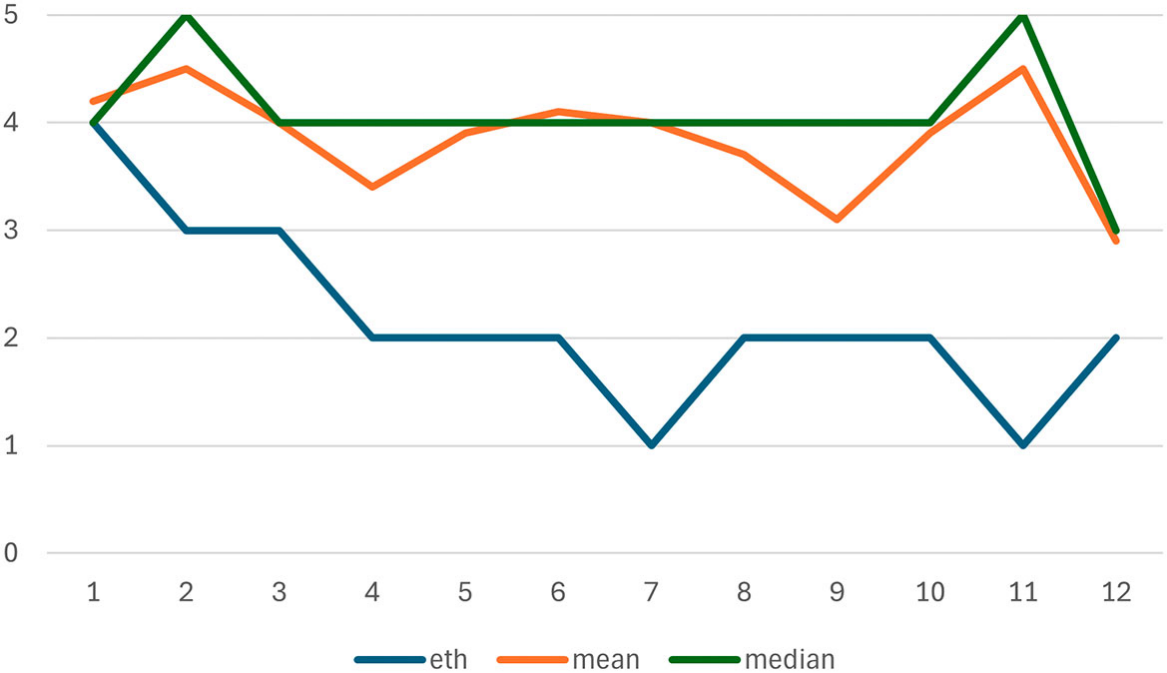
Comparing the ethicist’s estimate of the severity of 12 identified issues with the mean, and medianstakeholder estimates.

Figure 2 graphically depicts the differences between the ethicist performing the EIA and the mean, and median estimates of the respondents.

Comparing the highest and lowest scores in Figures 4, 5, 6 and 7, shows that there is a large variance between individual responses of participants in the XX Seminar on Nanotechnology, Society and Environment in Brazil on 18 October 2023. This suggests that stakeholder dialogue could be needed to address significant differences of opinions on the severity of specific issues. Stakeholder dialogue appears most urgent regarding issue 4 ( *Not sharing nanosafety data slows down the limiting of environmental impacts of nanomaterials*), and 9 ( *Researchers in resource poor settings could be disadvantaged if forced to share their data*), because the range between the highest and lowest scoring participant spans the full 5-point scale. Also regarding issues 6, 8 and 10 there appears to be large disagreement among the respondents on the severity of the issues (a 4-point difference between the lowest and highest scores). The apparent disagreement on the severity of issues 3, 5, 7, 11 and 12 is smaller but still substantial (3 points).

**Figure 3.**
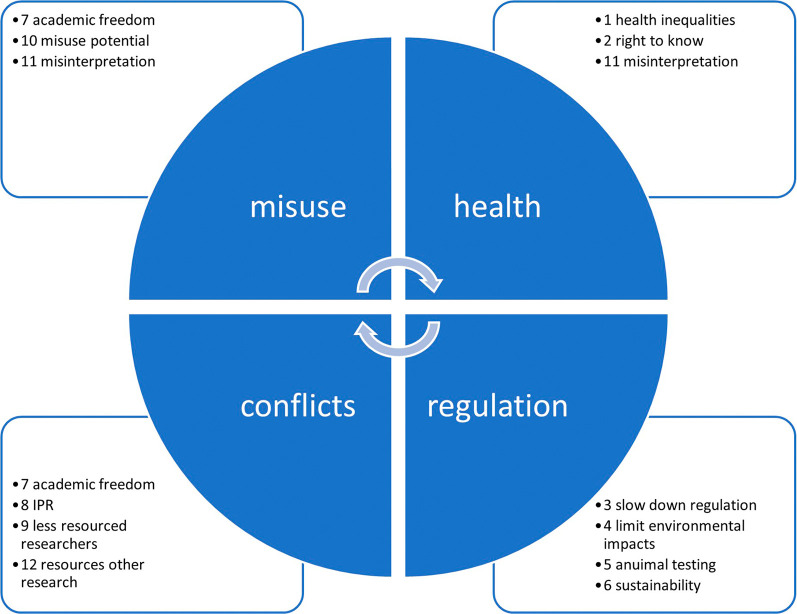
Clustering related ethical issues for stakeholder dialogue.

Since some issues are related, any dialogue should address a cluster including all of them. For example, issues 7, 10 and 11 are all related to misuse or misinterpretation of publicly-shared data. Likewise, issues 1, 2 and 11 are related to human health risks, and issues 3, 4, 5 and 6 address chemicals R&I regulations The interests of researchers are at stake in issues 7, 8, 9 and 12 (see Figure 3).

**Figure 4.**
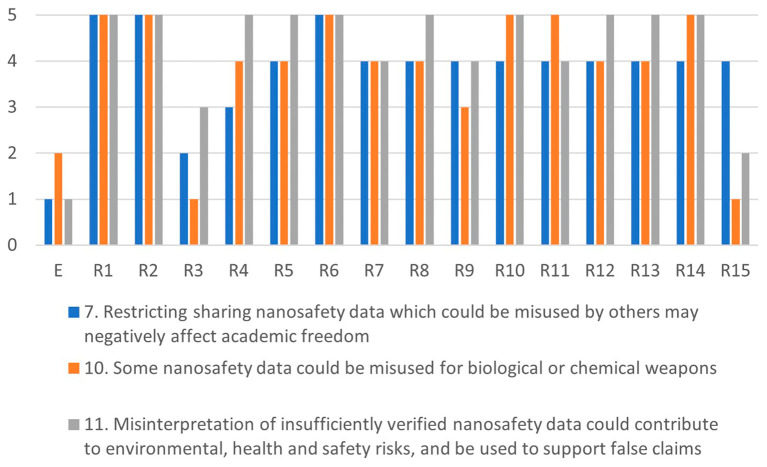
Differences in assessments between the ethicist (E) and individual stakeholders for the misuse-cluster.

**Figure 5.**
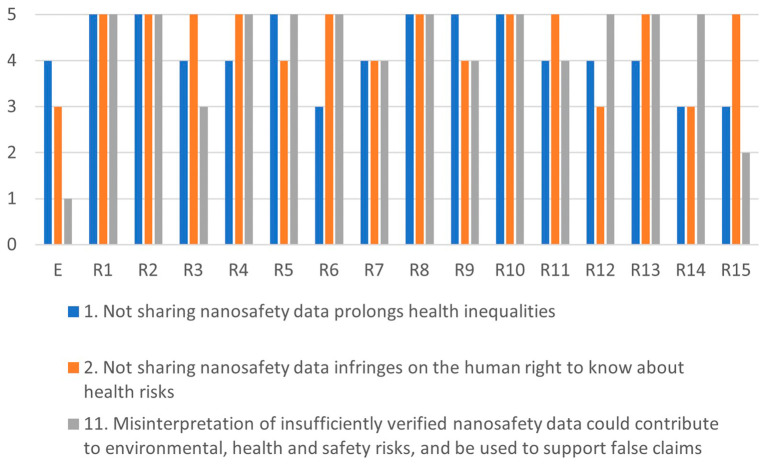
Differences in assessments between the ethicist (E) and individual stakeholders for the health-cluster.

**Figure 6.**
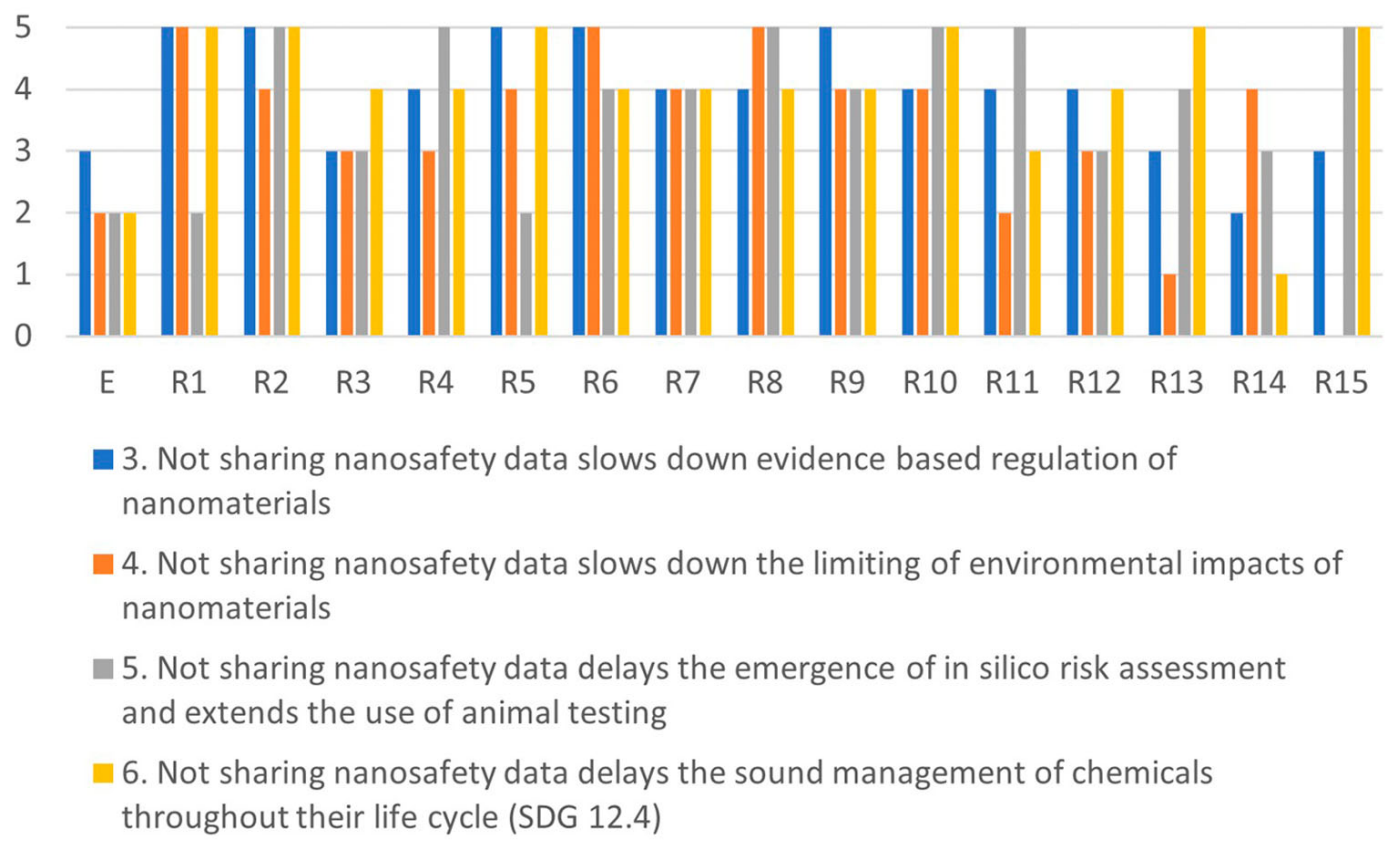
Differences in assessments between the ethicist (E) and individual stakeholders for the regulation-cluster.

**Figure 7.**
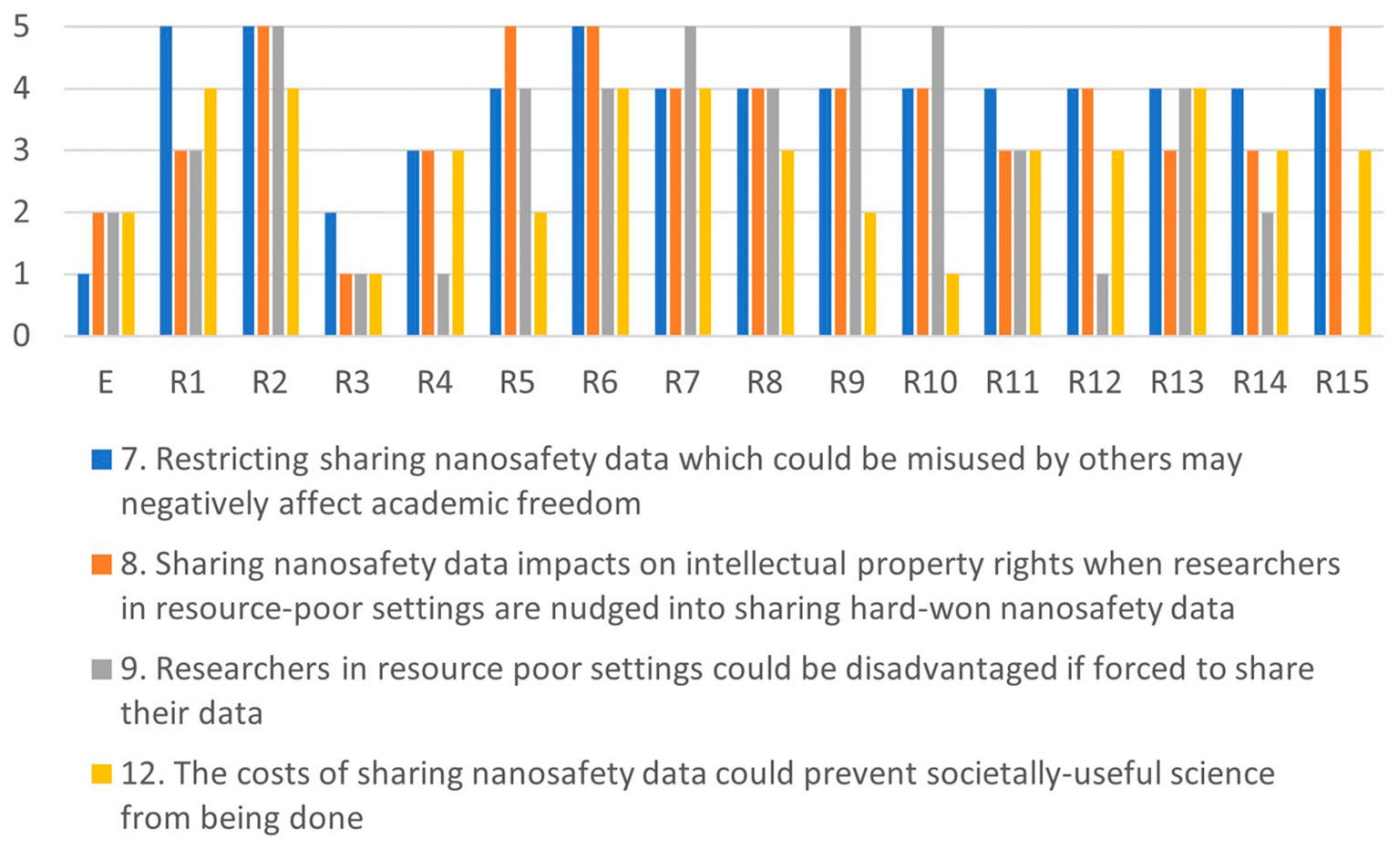
Differences in assessments between the ethicist (E) and individual stakeholders for the conflicts-cluster.

Figure 4– Figure 7 illustrate the differences in the assessments of the severity between issues related to misuse ( Figure 4), health ( Figure 5), regulation ( Figure 6) and conflicts ( Figure 7). More in-depth dialogue to explore underlying value conflicts and make them more explicit goes beyond the scope of this paper.

***Day-to-day concerns of data scientists.*** During the Bioinformatics in Action seminar at Maastricht University on 11 January 2024, participating data scientists raised two types of ethical concerns encountered in their day-to-day practice. Both issues, dual-use versus open access ( Text Box 2), and data quality of reused data ( Text Box 3), were related to the misuse cluster, while the latter was also related to the health cluster, in Figure 3.

Text Box 2: **Dual-use versus open access.**

The researchers were aware that national and EU rules for “Tackling R&I Foreign Interference” ( European Commission, 2022) are becoming more restrictive. According to the accompanying EC staff working paper, research organisations must take measures to prevent foreign states’ misuse of knowledge developed by the research organisations. Open science is explicitly addressed. Regarding curated research data sets, the publishing researcher must make sure that no sensitive or confidential data and metadata are published. In addition, review procedures for published data sets should also be developed, possibly including an assessment of impacts of accumulation of big data and artificial intelligence. Open FAIR data raises the same issues as machine-readable open data, while the risks of foreign interference are less obvious for restricted FAIR data. The increasing use of machine learning and artificial intelligence is expected to raise more concerns for foreign interference, which call for strengthened awareness of researchers ( European Commission, 2022, p19).

A specific example from the daily practice of the data scientists participating in the Bioinformatics in Action seminar concerns obstacles to their ongoing collaboration with Iranian colleagues. Researchers in Iran cannot presently access the new WikiPathways website ( www.wikipathways.org, Agrawal *et al.*, 2024) directly, but only the classic WikiPathways ( https://classic.wikipathways.org/, Ankley *et al.*, 2010), which is no longer further developed. While the national policies in some EU countries (e.g. in The Netherlands: https://english.loketkennisveiligheid.nl/) and the EC proposal for a “Council Recommendation on Enhancing research security in Europe” ( European Commission, 2024) call for balancing academic freedom and security on a case by case basis, decision making on access to WikiPathways is apparently beyond the reach of these scientists.

Text Box 3: **Data quality of Reused Data.**

The data scientists at Maastricht University also raised the issue of data quality of reused data. The R of FAIR addresses Reusability of data. To help ensure proper data quality, the researchers supplying data must correctly report specific metadata in addition to the results of their experiments, but this is not always the case. In their turn, the data scientists insert such published data in computer models to create Adverse Outcome Pathways to understand how toxic substances cause adverse outcomes (Reference: 10.1002/etc.34, van Rijn *et al.*, 2024). They digitise information on real experiments, and publish the results on WikiPathways, which are then reused by other people. In addition to data, hypotheses offering different opinions on how processes may occur are also published on WikiPathways. The end user of the data may not be sufficiently aware of the quality and relevance of the original data.

The data scientists were particularly concerned with data quality problems of using outdated data or data from retracted publications. The retraction problem covers the research integrity issues fraud and plagiarism, but even when the data are correct, the conclusions in the publication may be wrong. To make things worse, retracted articles are still cited a lot after they were retracted. This could be because of the retraction or despite this. Some such citations could be legitimate (e.g., criticism of the retracted work, or use of claims that are unrelated to the reasons for the retraction), but in many cases, citations to retracted articles are problematic. A related issue is that standardised criteria appear to be lacking for deciding when to delete outdated or wrong data and to determine who has the right or obligation to delete the data. This is an example of a ‘many hands problem’ posing barriers to responsible research and innovation (e.g., van de Poel *et al.*, 2012).

## Evaluation of ethical impacts

The identified ethical issues are discussed in the following subsections and analysed by applying relevant ethical theories and concepts. This evaluation is guided by online tools, and again results in a report on the evaluation of ethical impacts ( http://www.enaloscloud.novamechanics.com/riskgone/EIA/evethimpact/index.zul).

### Intellectual property

Moore & Himma (2022) define intellectual property (IP) law as protecting a content-creator’s interest in their ideas by assigning and enforcing legal rights to produce and control physical instantiations of those ideas.

At a theoretical level, Moore and Himma also discuss moral justifications for intellectual property: namely, personality-based, utilitarian, and Lockean, as well as numerous critics of intellectual property and systems of intellectual property protection. The decision to publish nanosafety data is under normal circumstances the prerogative of the creator or owner of the data. From a utilitarian perspective, nanosafety data should be shared if it contributes to greater good to a larger number of people than not sharing nanosafety data and *vice versa.* Whether data should be paid for or open access, and who can decide on secondary use of data after publication is subject to debate.

In practice, many open licenses (like Creative Commons, MIT/BSD/GPL) are actually based on IP law and protect the ownership of the creator/author rather than the publisher. However, the model to disallow people to reuse material unless they pay for it is a common business model for publishers, taking advantage of the need to ‘publish or perish’ of academic researchers.

Lawrence Lessig analysed the trend in increasing the duration of copyright protection in the USA. In his view, this trend is unjustly strengthening the market position of commercial companies holding copyright, while restricting the liberties of citizens who want to create derivative works and who hereby foster innovation ( Lessig, 2004).

The recent emergence of ChatGPT and its impacts on data protection and intellectual property raises new questions about what is fair use of open data. The EU recently adopted new legislation on copyright protection (European Union, 2019), including exceptions for “text and data mining for the purposes of scientific research” (article 3). This data should be stored in databases with restricted access. In addition, the Data Act (European Union, 2023), regulates the internal market for data generated by devices and shared online in the internet of things. Rights and obligations for data owners, companies and other users are specified.

During the Bioinformatics in Action seminar, a data scientist raised the issue that not a lot of nanosafety data are Findable online. Some knowledge is shared, but then data scientists learn about it much later. For example, scientists often share their most recent insights at conferences, yet, this knowledge might only appear several months or even years later in journals, which can delay the flow of peer-reviewed information on online platforms. Such delays may also disadvantage researchers who are unable or unwilling to travel to conferences (e.g., because of lack of resources, care duties, climate concerns) while offering a competitive advantage to researchers who do travel to conferences (who tend to already possess various advantages). In other words, relying on conferences for diffusion of knowledge may exacerbate inequalities. A colleague held a slightly different view, stating that the amount of available data is not the problem, but a lot is not shared publicly. Closed information is not sound, because it is closed. If data scientists openly describe that this compound is effective for this disease, companies cannot patent it anymore, and then no drug will be developed. More specifically, with respect to Covid-19, proteins may serve as potential drug targets, but privacy and economic concerns pose obstacles. For protecting the privacy of patients, technical solutions exist – instead of sharing the data, federated data can be analysed. Economic interests are a more intractable problem. New drug development is extremely expensive. If a company shares the data, competitors will be advantaged. Can data scientists develop something similar to the privacy protection measures, but for addressing economic concerns? For technical data, patents offer the solution, stimulating disclosure while granting exploitation rights to allow for a return on investment. Could the risk assessment data be shared in a similar form as a patent? That could be interesting for companies. However, generative AI like ChatGPT ignores protective constructs like ‘non-commercial’ clauses for open data.

### Social justice

The TRUST global code of conduct on research in resource-poor settings (2018) addresses unbalanced economic resources and the participation of people in countries in the Global South. The code identifies four leading values (fairness, respect, care, and honesty), that can foster greater social justice in research. Here, social justice means recognition of individuals’ mutual obligations to each other, including the obligation to create and maintain s *ystems* that facilitate equality of access and opportunity. Of course, in practice many people perceive that they have greater obligations to some people (e.g., to family members) than to the collective and/or institutions such as the nation-state may shape individuals’ beliefs about whom they are mutually obligated to. There are therefore trade-offs between a social justice approach and, for example, national-security concerns.

Without disregarding that caveat, we note that the social justice perspective highlights current unequal distributions of resources among different groups of researchers in the production of nanosafety data, including researchers working in countries in the Global South, as well as freelance researchers who do not benefit from institutional affiliations. The social justice imperative means that encouraging disadvantaged researchers to share their nanosafety data must go hand in hand with investments in their research infrastructure and facilities (and/or access thereto), creating a level playing field in the international competition for recognition and research careers.

### Public health ethics

Faden *et al.* (2022) differentiated public health ethics from biomedical ethics. While the latter targets the care for individual patients, the former is more concerned with statistical health at the level of populations. Public health ethics can be analysed from a justice perspective or from the perspective of the legitimacy of the state to restrict liberties to promote public health. Voluntarily sharing nanosafety data to contribute to regulations protecting public health arguably contributes to more justice, across several ethical traditions (e.g., promoting the common good, or the greatest good to the largest number of people). Obliging researchers to share their nanosafety data should be justified on a case-by-case basis, balancing the expected gain in public health with the restrictions on the liberties of the researcher. In different regulatory regimes, such balancing acts have been resolved in different ways.

### Dual-use and misuse

The risk that states, terrorists or criminals misuse scientific knowledge and technologies for biological or chemical weapons are analysed from a deontological ethics perspective. Governments and research managers impose laws and professional codes of conduct on researchers working with dual-use materials, technologies or related knowledge. How to balance state and civil security and academic and entrepreneurial freedom in each particular case is a key issue in the debate. Some nanosafety data could be misused for developing chemical weapons, as addressed by Bezuidenhout and Morrison (2016). Several EU countries are engaging with the boards of Higher Education Institutes to develop risk assessments and guidelines for complying with recent research security regulations. The European Commission has proposed a recommendation to harmonize national regulations ( European Commission, 2024). Balancing the values of academic freedom and security is at the core of these policies.

### Environmental and animal ethics

Brennan and Lo (2022) explained that protection of the environment is perceived differently from the perspective of the main ethical traditions. From a consequentialist perspective, data-sharing as such is not ethically right or wrong, but the expected overall resulting impact on the environment should be optimised. On the one hand, if a community agrees to share data, then all kinds of existing data should be shared, including data that shows that a certain nanomaterial is toxic to the environment and data that shows that this nanomaterial is not harmful to the environment. It is important to allow for discussions of contradictory scientific evidence, which is only possible if all kinds of data are shared. On the other hand, the costs of sharing should be taken into account as well. If, for instance, a requirement was imposed that all scientific data should be kept on publicly accessible servers indefinitely, those servers cost money to set up and operate, and have a significant footprint in terms of energy and water usage, noise and thermal pollution, and other environmental factors.

From a deontological perspective, sharing data on environmental tests of nanomaterials can be right or wrong depending on moral considerations, disregarding the eventual impacts on the environment. For example, not killing or harming species is intrinsic good. Whether sharing nanosafety data is right or wrong depends on prioritising either the need to protect the environment from harmful nanomaterials, or protecting the intellectual property of the data owners.

Whether using animals in nanosafety testing is right or wrong depends on balancing the intrinsic worth of these animals with the intrinsic worth of the human beings and environment to be protected by regulations informed by the collected data.

### Balancing ethical risks and benefits

To summarise, sharing nanosafety data is likely to bring more significant benefits than raise ethical problems. On the beneficial side, strong impacts on public health and related values such as common good and equal distribution of risks and hazards are expected, as well as moderate benefits to the environment and in the long term minor benefits to animals, because of the reduced need for animal testing. On the detrimental side, moderate impacts on intellectual property of data owners and social justice of researchers in resource-poor settings are expected. Sharing some nanosafety data may introduce moderate ethical issues and the use of animals in nanosafety studies has negative impacts on animal ethics in the short term (See Table 2).

**Table 2.** Balancing ethical risks and benefits of sharing nanosafety data. 1 = minor; 2 = moderate; 3 = medium; 4 = high; 5 = extreme.

Degree of violation	Identified principle or value	Degree of benefit
2	intellectual property	0
2	social justice	0
0	public health	4
2	dual-use	0
0	environmental ethics	2
1	animal ethics	1

Remediation measures to address the foreseen ethical impacts of sharing nanosafety data is not in all cases unproblematic. Some trade-offs between conflicting principles or values must first be resolved. In general, the main trade-off related to sharing nanosafety data is between public health and social justice. When the nanosafety data could be misused by others, a trade-off between security and freedom must be addressed, and when animal experiments are performed, there is a trade-off between environmental and animal ethics. In the next section, recommendations for remediation of these trade-offs will be discussed.

## Recommendations for remediation

Recommendation for remediating the identified ethical issues and value conflicts were drafted, guided by online tools in the RiskGONE cloud platform ( http://www.enaloscloud.novamechanics.com/riskgone/EIA/remedialactions/index.zul). Ten participants in the XX Seminar on Nanotechnology, Society and Environment commented on these recommendations in an anonymous online survey. In addition, the preliminary findings and recommendations were discussed with data scientists of Maastricht University during the Bioinformatics in Action seminar on 11 January 2024. The discussed issues related to retraction of published data and reusing data which has not been updated, dual-use and international cooperation subject to export control regulations, and protecting economic interests while publishing open data. The received comments are incorporated in the following discussion of the recommendations.

### Societal and policy recommendations

Several societal recommendations for governments and stakeholders involved in risk governance of nanomaterials and open data initiatives were found in the literature.

***Trade-off between public health and social justice.*** To address the trade-off between public health and social justice, Bezuidenhout (2017) proposed key ways to address “binds of pace” in open data discourse, including an expanded understanding of laboratory equipment and research speed to include all aspects of the research environment. This should be combined with better engagement with scientists from countries in the Global South regarding these challenges and the adoption of frugal or responsible design principles in future open data initiatives. Six stakeholders agreed or considered this important. One stakeholder did not agree. One respondent commented “more resources would be ideal, both human and material”, while another commented: “If it were possible to harmonize the researchers and make them work together, it would make it easier to understand nanotechnologies, because two heads are better than one”.

In addition, the inequalities between resource-rich and resource-poor researchers can be remediated through voluntary sharing of nanosafety data, imposing more responsibility on richer rather than poorer research managers and scientists. If, for instance, FAIR or Plan S were imposed on researchers from the Global South (e.g., by journals or collaborators in the Global North) then the costs to those researchers would need to be evaluated carefully and counterbalanced in some way.

***Trade off dual-use and open data.*** To reconcile the responsibilities of life scientists posed by the open data and dual-use frameworks, Bezuidenhout and Morrison (2016) suggested focusing regulatory approaches more on the everyday practices of laboratory scientists and less on abstract conceptions of data. Regulators would need to communicate with researchers to understand these everyday research practices. Seven stakeholders expressed agreement with focusing approaches more on everyday research practices, using different words. Also, one partially agreed, and one commented: “It will make it easier to understand nanoparticles”.

***Trade-off between economic interests and open data.*** Technical as well as legal solutions were proposed by participants in the Bioinformatics in Action Seminar, to balance economic interests of innovative companies and the open data requirements of the nanosafety community. A political recommendation addressing this issue is to change the legal context to protect the nanosafety data and still make it open, mirroring patent law. Researchers can stimulate politicians to change the laws.

### Organisational recommendations

Organisational recommendations are proposed for the management of research organisations producing the nanosafety data. They are recommended to raise awareness of research integrity principles and train researchers in best practices ( Kretser *et al.*, 2019). Nine respondents agreed in various wordings.

***Understand everyday research practices.*** How research organisations can contribute to better understanding of everyday research practices is illustrated by a pilot in the framework of the e-NanoMapper project. Requirement analysis was performed by a consultant, observing researchers during a day in the laboratory. The consultant took notes, collected artefacts (significant objects) during this participant observation and clustered the anonymised notes. This answered the question: “what should the data platform do to help researchers do their work more efficiently?” This is based on the rapid contextual design approach ( Holtzblatt *et al.*, 2004). In addition, STS researchers who do lab-ethnography are often embedded for several years in scientific teams and gain detailed insights into everyday scientific practices. Lab-ethnographers can therefore give elaborate overviews over the flow of material and information in the laboratory ( Latour & Woolgar, 2013) and identify implicit assumptions, fears, and dynamics in scientific collaborations, which can help to work through existing conflicts and implement reforms ( Nelson, 2021).

***TRUST code.*** Managers of research organisations and nanosafety researchers collaborating with researchers in countries in the Global South and freelance researchers are recommended to implement the Global Code of Conduct for research in resource-poor settings (2018). This code includes practical advice to adhere to core values of fairness, respect, care, and honesty. Six respondents expressed some kind of agreement. One commented: “I think this should follow a pattern”, one sceptically reflected: “Great, if only there was a perfect world where everyone lived in harmony, but the big problem is the creation of biological weapons.” One person stated: “I don’t have the information to give an opinion”.

***Animal testing.*** Researchers using animal testing to produce nanosafety data are recommended to comply with the 3Rs ( Zurlo *et al.*, 1996): reduce the number of tests, refine the testing procedure, and replace animal testing with other methods ( *in vitro, in silico*). Seven stakeholders agreed in various wordings, while one expressed scepticism: “now that would be great”. One person agreed partially: “It will not necessarily be possible to substitute animal tests”, and another thought: “You can keep testing on animals for a better approximation of results with humans”.

***Data quality of reused data.*** Data scientists could address the data quality issues of WikiPathways by raising the awareness among users of issues like the fact that data may not be up to date, retracted data may be used, and that hypotheses are placed on WikiPathways in addition to data. However, data scientists may not be the best placed to address the retraction issues, calling for interdisciplinary collaboration. For example, for library scientists, it is easier to look at retractions, and investigate why it was retracted. They are also more aware of prior literature, and of negative citations. To address the connected issues of indefinitely storing obsolete data on energy intensive servers, and the inadvertent loss of data stored on old data storage devices, data stewards could develop practical standard approaches for sound data curation.

***Trade off economic interests and open data.*** In addition to legal measures, technical solutions were proposed by participants in the Bioinformatics in Action Seminar, to balance economic interests of innovative companies and the open data requirements of the nanosafety community. A technical recommendation could be to learn from technical solutions for data protection, where federated data can be analysed, instead of sharing the data. Data scientists are challenged to develop similar solutions to address economic concerns.

## Conclusions

This Ethical Impact Assessment has identified the following ethical issues and value conflicts at stake in decision making on openly sharing nanosafety data needed for risk governance of nanomaterials: intellectual property, social justice, public health, dual-use, environmental ethics and animal ethics. Ethical principles and values supporting openly sharing nanosafety data cluster around health and environmental protection, fostering evidence-based regulation and reducing further delays in achieving the long overdue Sustainable Development subgoal SDG 12.4: “the achievement of the sound management of chemicals throughout their life cycle so that by the year 2020, chemicals are produced and used in ways that minimize significant adverse impacts on the environment and human health.” Other ethical principles and values call for imposing some restrictions on openly sharing nanosafety data, including dual use and misuse concerns and equal participation in science by disadvantaged researchers. To balance these conflicting ethical principles and values, several issues can be addressed by implementing recommendations for remedial actions discussed above. Only in some cases will it be possible to conclude that nanosafety data in general should not be published, for example if the data are not owned by the person publishing it, who has not obtained permission from the data owner or is not entitled to publish it for other reasons such as legal obligations. In other cases, the decision to publish data or not should be taken on a case-by-case basis. Furthermore, the timing and form of publication of the data can also vary, again depending on the specific case at hand. The strong differences of opinions between the ethicist and the average respondent on the severity of twelve identified ethical issues, and the wide range in assessments of several issues among the respondents, calls for follow-up stakeholder dialogue. Such follow-up dialogue could be organised in four clusters: related to a) health, b) the potential for misuse or misinterpretation of nanosafety data, c) impacts on chemicals regulation, and d) balancing the interests of distinct groups of researchers.

On a methodological note, the systematic difference between the ethicist’s estimates of severity and the average stakeholder’s estimates suggests two different uses of the five-point scale, (i) semi-quantitative: to reach a common understanding of the severity of each identified issue, or (ii) comparative: to identify issues for stakeholder dialogue delving into underlying value conflicts. In the semi-quantitative mode, the professional ethicist can use the scale to identify and prioritise issues subject to Ethical Impact Assessment, placing the identified issues in the context of ethical knowledge about the severity of ethical issues related to science and technology, and other human activities. Before involving multiple experts or stakeholders in corroboration of these estimates, the interpretation of scales should be discussed thoroughly. In cases of large discrepancies, the evaluation step can be re-iterated a second time (or third and more times) by taking into consideration the evaluation results of the previous rounds, if deemed relevant.

In the comparative mode, the interpretation of the scale should be left to the participating stakeholders, who can use the scale more as a relative assessment tool to compare the severity of distinct identified ethical issues. In the subsequent dialogue, their arguments for their estimated severity should be explicitly discussed, as well as their views on the issue at stake.

The emerging application of AI in generating and analysing nanosafety data appears to raise unprecedented issues, to be addressed in follow-up research using foresight methods.

A reviewer proposed to explain how ethics could be embedded in FAIR data infrastructures. However, ethics is more about balancing ethical values and principles through recommended actions including technical, organisational, societal and political measures (e.g., ethics by design, training staff in research ethics, public dialogue, changing legislation). For nanosafety researchers wishing to address ethical issues of their research, the Ethical Impact Assessment tools developed in RiskGONE ( https://www.enaloscloud.novamechanics.com/riskgone/EIA/), as well as the EU’ s ethics guidelines for Horizon Europe proposals ( https://ec.europa.eu/info/funding-tenders/opportunities/docs/2021-2027/common/guidance/how-to-complete-your-ethics-self-assessment_en.pdf) and the UNESCO Ethical Impact Assessment guidelines for responsible AI ( https://www.unesco.org/ethics-ai/en/eia) offer useful guidance.

Another reviewer called for clarification of the reproducibility of this Ethical Impact Assessment methodology to other cases of sharing data. While the online tools developed in RiskGONE are mainly focused on nano-related research, they are based on the prestandard CEN CWA 17145–2: 2017 (E): ( CEN, 2017), which is applicable to any R&D&I project raising ethical issues. Also, the above-mentioned UNESCO Ethical Impact Assessment methodology for Responsible AI is a similar approach addressing other data-sharing related cases.

## Review of EIA

The prescribed review of the final manuscript reporting on the EIA will be performed by external peer reviewers.

## Ethics policies

Data protection: no personal data has been collected, processed or stored in the research reported in this article. Human participation was limited to professional researchers participating in scientific conferences and a seminar. Participation was voluntary. The research was part of a H2020 project which passed ethics screening. Explicit approval by an ethical committee of the human participation in the research reported in this article was not necessary in accordance with Section E in the CODE OF ETHICS FOR RESEARCH IN THE SOCIAL AND BEHAVIOURAL SCIENCES INVOLVING HUMAN PARTICIPANTS. As accepted by the Deans of Social Sciences in the Netherlands, 23 mei 2018: Enschede: University of Twente. https://www.utwente.nl/en/bms/research/forms-and-downloads/code-of-ethics-for-research-in-the-social-and-behavioural-sciences-dsw.pdf

## Ethics and consent

Since no personal data were collected, stored or processed, consent by data owners was not necessary. Tacit consent by the participating researchers in the research was sufficient in accordance with section E in the CODE OF ETHICS FOR RESEARCH IN THE SOCIAL AND BEHAVIOURAL SCIENCES INVOLVING HUMAN PARTICIPANTS. As accepted by the Deans of Social Sciences in the Netherlands, 23 mei 2018: Enschede: University of Twente. https://www.utwente.nl/en/bms/research/forms-and-downloads/code-of-ethics-for-research-in-the-social-and-behavioural-sciences-dsw.pdf

## Data availability statement

The raw data of the online survey among participants in the XX Seminar on Nanotechnology, Society and Environment in Brazil and online, on 18 October 2023 is available at.

Repository name: ZENODO.

Title of project: Ethical Impact Assessment of sharing nanosafety data: raw data of the online survey among participants in the XX Seminar on Nanotechnology, Society and Environment in Brazil and online, on 18 October 2023.

DOI (Citation): https://zenodo.org/records/13848851 ( Malsch, 2024).

This project contains the following underlying data: Ethical Impact Assessment of sharing nanosafety data: raw data of the online survey among participants in the XX Seminar on Nanotechnology, Society and Environment in Brazil and online, on 18 October 2023.

Name and description of each file: One excel sheet includes the responses to the online survey on Ethical Impact Assessment of sharing nanosafety data of participants in the XX Seminar on Nanotechnology, Society and Environment in Brazil and online, on 18 October 2023, in Portuguese, and the other the translated responses in English.

SurveyrenanosomaEnglishraw.xlsx

Surveyrenanosomaportugueseraw.xlsx

Details of license: Data are available under the terms of the Creative Commons Attribution 4.0 International.
