# Supplementary material for: Ethical Impact Assessment of Sharing Nanosafety Data
Source: Open Res Eur. 2026 Apr 6;4:237. Originally published 2024 Oct 25. [Version 2] doi: 10.12688/openreseurope.18345.2 (PMC13062766; doi:10.12688/openreseurope.18345.2)
Supplement: Peer review history

# Reviewer report: Reviewer response for version 2

**DOI:** 10.21956/openreseurope.25385.r71959

**Published:** 2026-04-14

**Authors:** Benjamin Punz

**Affiliations:** 1 Paris Lodron University Salzburg, Salzburg, Austria

I would like to congratulate the authors on this very interesting and thought-provoking article addressing the ethical considerations associated with sharing nanosafety data based on the results of an ethical impact assessment. I have a number of minor comments that I hope will be helpful in further strengthening the manuscript.

- Minor comment 1: In the introduction, the reference cited when FAIR data is first mentioned appears to be incorrect. “(Nature, 2016)” should likely be replaced with “(Wilkinson et al., 2016)”.
- Minor comment 2: Figure 1 could benefit from further refinement to clarify the origin and meaning of the presented information. Based on the text, it was not entirely clear how the figure relates to the outputs of the RiskGONE Ethical Impact Assessment screening tool. In particular, the scale ranging from -5 to 5 is not explained, and certain categories (e.g., Military Dual Use, Privacy) mentioned in the legend do not appear in the figure.
- Minor comment 3: The figure captions are generally quite brief. Expanding the descriptions would improve interpretability, as the current captions provide limited additional context beyond the figures themselves.
- Minor comment 4: Axis labels are missing in several figures (Figures 1, 2, 4, 5, 6, and 7). Adding these would improve clarity and readability.
- Minor comment 5: There is a typographical error on page 7: “Example,s of this web of prevention are:…”. This should be corrected.
- Minor comment 6: At the beginning of the section “Ethical impacts of not sharing nanosafety data,” the first sentence refers to a scale ranging from 1 to 4 on a 5-point scale. As a reader not deeply familiar with this framework, it was initially unclear why the full range (including 0 and 5) was not addressed. This is clarified later in the text (after Table 1), but introducing this explanation earlier would improve readability and comprehension.
- Minor comment 7: In the caption of Figure 2, there appears to be a missing space or separator in “medianstalkeholder,” which should be corrected.
- Minor comment 8: In Figures 4 - 7, stakeholders are abbreviated as “R.” It would be helpful to clarify this abbreviation, as it is not immediately intuitive.
- Minor comment 9: Consider grouping Figures 3–7 more closely together and ensuring they are clearly introduced in the text. The current description could be streamlined and clarified, for example by restructuring the paragraph that links specific issues to the respective figures to improve readability and flow.
- Minor comment 10: In the “Environmental and animal ethics” subsection, it may be valuable to highlight the broader issue of publication bias. In many cases, only studies demonstrating significant effects are shared, whereas no-effect studies remain unpublished. Including such data would be highly beneficial for the community, as it would help complete datasets and prevent redundant research efforts aimed at addressing already explored but unpublished experimental gaps.

If any, are all the source data and materials underlying the results available?

Yes

Is the work original in terms of material and argument?

Yes

Is the work clearly and cogently presented?

Yes

Is the argument persuasive and supported by evidence?

Yes

Does the research article contribute to the cultural, historical, social understanding of the field?

Yes

Does it sufficiently engage with relevant methodologies and secondary literature on the topic?

Yes

Reviewer Expertise:

Nanoscience, SSbD, Biotechnology

I confirm that I have read this submission and believe that I have an appropriate level of expertise to confirm that it is of an acceptable scientific standard.

# Reviewer report: Reviewer response for version 2

**DOI:** 10.21956/openreseurope.25385.r71919

**Published:** 2026-04-08

**Authors:** Ajay Vikram Singh

**Affiliations:** 1 German Federal Institute for Risk Assessment (BfR), Berlin, Germany

The authors have addressed the concerns I raised in the previous review. The revisions have improved the clarity and quality of the manuscript. I recommend that the paper be accepted. I approve indexing.

If any, are all the source data and materials underlying the results available?

Yes

Is the work original in terms of material and argument?

Partly

Is the work clearly and cogently presented?

Partly

Is the argument persuasive and supported by evidence?

Partly

Does the research article contribute to the cultural, historical, social understanding of the field?

Yes

Does it sufficiently engage with relevant methodologies and secondary literature on the topic?

Partly

Reviewer Expertise:

Regulatory Toxicology, in silico NAMs, Experimental Toxicology

I confirm that I have read this submission and believe that I have an appropriate level of expertise to confirm that it is of an acceptable scientific standard.

# Reviewer report: Reviewer response for version 1

**DOI:** 10.21956/openreseurope.19823.r56284

**Published:** 2025-08-14

**Authors:** Ajay Vikram Singh

**Affiliations:** 1 German Federal Institute for Risk Assessment (BfR), Berlin, Germany

The manuscript titled "Ethical Impact Assessment of Sharing Nanosafety Data" addresses an important and timely gap in the nanosafety field. After carefully reviewing, here is my evaluation report with detailed, feedback to help improve the manuscript’s scientific quality, clarity, and overall impact:

 Conceptual and Methodological Clarity- The paper addresses a critically under explored intersection—ethics in nanosafety data sharing—with a structured framework drawing from the Ethical Impact Assessment (EIA) methodology. The methodological description of the EIA framework (particularly how it was adapted or modified for nanosafety) lacks operational clarity. While it claims to adapt existing frameworks, it should explicitly state how each phase was applied, possibly with a flow diagram or schematic. This would also help distinguish it from standard ELSA approaches. Introduce a visual schematic of the ethical assessment pipeline adapted for nanosafety data—this would clarify the novelty and practicality of the proposed process. Add a seminal work DOI: https://doi.org/10.1166/jnn.2014.8747 with the sentence ´The physicochemical parameters of engineered nanomaterials involve intrinsic properties (e.g., shape, porosity, and bandgap), extrinsic properties (e.g., concentrations and surface interactions with biomolecules), and composition aspects (e.g., charge, hydrophobicity, and coating)´ to update the bibliography.

 Terminology and Definitions- Some terms (e.g., "social sorting," "epistemic justice," "anticipatory ethics") are introduced without sufficient contextual definition for readers from a broader regulatory or toxicology background. Include a concise glossary or a boxed section defining key ethical-philosophical concepts used throughout the manuscript.

 Depth of Ethical Analysis - While ethical concerns are mentioned (e.g., dual-use risk, data misuse, epistemic bias), the discussion lacks concrete use cases or hypothetical scenarios to ground these issues in real-world nanosafety contexts. Include 1–2 short case vignettes that illustrate how ethical dilemmas in data sharing might emerge in real nanosafety research (e.g., toxicity data being misinterpreted, or withheld due to commercial IP concerns).

 Integration with Data Infrastructures- The discussion on FAIR principles and the data-sharing challenges is timely. However, it lacks detail on how ethical assessments could be embedded into digital infrastructures (e.g., via metadata, access protocols, or AI-driven monitoring). Expand on how ethical considerations can be built into nanosafety databases like NanoCommons or eNanoMapper—perhaps using a table comparing ethical readiness or governance levels.

 Balance of Critical vs. Normative Tone- In some parts, the text reads as advocacy (e.g., promoting openness and inclusivity), which is valid, but the paper would benefit from greater critical balance. Discuss the potential trade-offs—e.g., between open access and intellectual property, or between transparency and national security—more explicitly. With the sentence ´The goal of *in silico *assessment tools is to investigate which physicochemical parameters of engineered nanomaterials could lead to toxic mechanisms in a given biological environment ´, cite Bansod G, et al., 2025 [Ref 1] a recent report to make literature up to date.

 Table 1 – Ethical Issues in Nanosafety Data Sharing- Well-structured list summarizing key issues. Consider adding a column for “Potential Mitigation Strategies”—this would make the table more action-oriented. Clarify the source or rationale for issue selection—are these from literature, interviews, or the authors' expert judgment?

 Figure 1 – Phases of Ethical Impact Assessment- Clean, logical progression from problem identification to recommendation. The visual design is too text-heavy; use color or icons to distinguish between stages. Add examples of methods or stakeholders involved at each phase (e.g., Phase II: Stakeholder mapping, surveys). Consider relabeling “Implementation” to “Translation & Governance” for clarity.

 Minor Comments- Grammar and style are generally strong, but occasional phrasing like “normative tools for ethical governance” could be simplified or clarified. Consistency in references to nanoinformatics tools and databases (e.g., eNanoMapper, NanoCommons) could be improved.

 In general- Deepen the methodological description of their ethical assessment framework,

- Provide practical examples or case scenarios,
- Embed more actionable elements into tables/figures, and
- Balance advocacy with critical ethical trade-offs.

If any, are all the source data and materials underlying the results available?

Yes

Is the work original in terms of material and argument?

Partly

Is the work clearly and cogently presented?

Partly

Is the argument persuasive and supported by evidence?

Partly

Does the research article contribute to the cultural, historical, social understanding of the field?

Yes

Does it sufficiently engage with relevant methodologies and secondary literature on the topic?

Partly

Reviewer Expertise:

Regulatory Toxicology, in silico NAMs, Experimental Toxicology

I confirm that I have read this submission and believe that I have an appropriate level of expertise to state that I do not consider it to be of an acceptable scientific standard, for reasons outlined above.

## References

[1] : Decoding Tattoo and Permanent Makeup Pigments: Linking Physicochemical Properties to Absorption, Distribution, Metabolism, and Elimination Profiles Using Quantitative Structure–Activity Relationship (QSAR)‐Based New Approach Methodologies (NAMs). *Advanced Intelligent Discovery* .2025; 10.1002/aidi.202500027 10.1002/aidi.202500027

# Response

**Published:** 2026-03-16

**Authors:** Neelina Hermina MALSCH

Dear Reviewer, Thank you for your extensive comments on our paper. While some of your comments seem to relate to another paper, we tried to respond to all your comments and suggestions to the best of our knowledge, as follows.

**Conceptual & methodological clarity: **the methodology was explained more in depth in an earlier paper. The reference to this is included more explicitly.

**Terminology & definitions: **The terms you mention are not used in this paper. Perhaps you are referring to another publication? In any case, we included some clarifications of terminology we did use, e.g., of social justice, and stakeholder engagement.

**Depth of ethical analysis: **The case of international collaboration with Iranian researchers is a clarification of the real-world case. We also added more extensive discussion of the impacts of closed research, comparing pharmaceutical companies and nanosafety researchers. More in depth ethical analysis goed beyond the scope of this paper.

**Integration with data infrastructures: **In the conclusions, we added a paragraph explaining how to embed ethics in digital infrastructures, referring to the EU Horizon Europe self-assessment, and UNESCO Ethical Impact Assessment of AI methodology. Just adding an ethics readiness box to FAIR digital infrastructures is not feasible, because ethical dilemmas call for balancing conflicting values and engaging different kinds of stakeholders in remediation.

**Table 1:** the sources of each of the 12 identified issues is explicitly mentioned in the text of the article. Adding a column on potential mitigation strategies here does not really fit in this table comparing the ethicists’ estimates and mean and medium stakeholder responses.

**Figure 1:** we moved the text to below the figure. Balance advocacy with critical ethical trade-offs: some sentences are added throughout the text. We added the reference you suggested.

# Reviewer report: Reviewer response for version 1

**DOI:** 10.21956/openreseurope.19823.r45583

**Published:** 2024-11-11

**Authors:** Siviwe Bangani

**Affiliations:** 1 Stellenbosch University, Stellenbosch, Western Cape, South Africa

Dear Editors,

 I must state upfront that I only have a rudimentary knowledge of the field of nano-safety, so my comments will lean towards data sharing in general rather than on the field itself. 

 The authors are congratulated for tackling the topical issue of ethics in data sharing. In my opinion, the article is well-written and the methodologies are novel and well-explained. I particularly liked the balancing act of  highlighting the dis/advantages of sharing data pertaining to nano-safety. Clear remedial actions are provided to offset or minimise the disadvantages. An attempt is made to link the work to SDG 12, specifically target 4 of this SDG but one is left with a sense that this important observation could have been covered in the conclusion. It was not clear to me if the methodological approach used in the study could be replicated in other environments. Perhaps the authors need to clearly articulate whether their methods are unique to this specific discipline or if they have the utility to be applied to data sharing in other science disciplines. Besides those two minor observations, I am happy that this work has been published.

If any, are all the source data and materials underlying the results available?

Yes

Is the work original in terms of material and argument?

Yes

Is the work clearly and cogently presented?

Yes

Is the argument persuasive and supported by evidence?

Yes

Does the research article contribute to the cultural, historical, social understanding of the field?

Yes

Does it sufficiently engage with relevant methodologies and secondary literature on the topic?

Yes

Reviewer Expertise:

Library and Information Science; Bibliometrics, Open Access; Community Engagement; Sustainable Development Goals; African Languages.

I confirm that I have read this submission and believe that I have an appropriate level of expertise to confirm that it is of an acceptable scientific standard.

# Response

**Published:** 2026-03-16

**Authors:** Neelina Hermina MALSCH

Dear Reviewer, Thank you for your encouraging comments and suggestions for improvement. We added a statement on the relation of our paper with SDG 12.4 and also references to the original CEN prestandard on ethical impact assessment and UNESCO ethical impact assessment methodology on responsible AI as examples of broader applicability of the methodology to other cases.
